# Nutritional status and quality of life in adults undergoing allogeneic hematopoietic stem cell transplantation

**DOI:** 10.1007/s12185-022-03351-7

**Published:** 2022-05-09

**Authors:** Marco Cioce, Stefano Botti, Franziska Michaela Lohmeyer, Eugenio Galli, Marinella Magini, Alessandra Giraldi, Paola Garau, Danilo Celli, Maurizio Zega, Simona Sica, Andrea Bacigalupo, Valerio De Stefano, Ivan Borrelli, Umberto Moscato

**Affiliations:** 1 Department UOC SITRA, Fondazione Policlinico Universitario Agostino Gemelli IRCCS, Largo Agostino Gemelli 8, 00168 Rome, Italy; 2 Haematology Unit, Oncology and Advanced Technology Department, Azienda USL-IRCCS Reggio Emilia, Reggio Emilia, Italy; 3 Scientific Directorate, Fondazione Policlinico Universitario Agostino Gemelli IRCCS, Rome, Italy; 4 Department of Hematology, Fondazione Policlinico Universitario Gemelli IRCCS, Universita’ Cattolica del Sacro Cuore, Rome, Italy; 5 UOC Clinical Nutrition, Fondazione Policlinico Universitario Agostino Gemelli IRCCS, Rome, Italy; 6 Faculty of Medicine and Psychology, Università “La Sapienza”, Rome, Italy; 7 Department of Life Sciences and Public Health, Università Cattolica del Sacro Cuore, Rome, Italy; 8 Department of Health Science of Woman and Child and Public Health-Occupational Health and Hygiene, Area-Fondazione Policlinico Universitario A. Gemelli IRCCS, Rome, Italy

**Keywords:** Allogeneic hematopoietic stem cell transplantation, Nutritional status, Malnutrition, Nutritional support, Quality of life

## Abstract

Although the effects of malnutrition on morbidity and mortality in adult patients undergoing allogeneic hematopoietic stem cell transplantation are clear, the relationship with quality of life (QOL) is less clear. The purpose of this study was to assess the relationship between malnutrition and QOL. A prospective observational study was conducted in 36 adult patients undergoing allogeneic hematopoietic stem cell transplantation. Adapted criteria of the Global Leadership Initiative on malnutrition have been used for the diagnosis of malnutrition in clinical settings. A cancer linear analog scale was used to assess QOL. Overall QOL at 14 days after allogeneic hematopoietic stem cell transplantation was 37.1 (95% CI 2.9–45.39) in patients without severe malnutrition, versus 16.0 (95% CI − 6.6 to 38.6) in patients with severe malnutrition (*p* = 0.05). At discharge, it was 48.0 (95% CI 38.4–57.6) versus 34.0 (95% CI 4.1–63.9) (*p* = 0.27). The results of our study suggest that patients with severe malnutrition at discharge tend to have worse QOL. A larger cohort of patients is required to confirm this hypothesis.

## Introduction

The allogeneic hematopoietic stem cell transplantation (allo-HSCT) is a standard therapy for a variety of malignant and not malignant hematology diseases including leukemia, lymphomas and other myelo-proliferative syndromes [1]. Although life expectancy is increasing, treatment remains associated with a series of complications related to the conditioning regime and immune-reactivity of infused cells. Among the most frequent, which can arise in the immediate post transplantation period accompanying the patient for several months, alterations of the nutritional status have been demonstrated [2–5], which are characterized by insufficient intake or uptake of nutrition [6]. Factors or conditions related to allo-HSCT may induce a significant reduction in oral food intake and/or intestinal malabsorption, such as total body irradiation, high-dose chemotherapy, steroids and immune-suppressors use, anorexia, dysgeusia, mucositis, nausea, vomiting, diarrhea, acute Graft-versus-Host Disease (aGvHD) and infections [7]. Inadequate nutrient intake or impaired uptake is often associated with weight loss. In the most serious forms, when the reduced food intake lasts longer than 7 days or if the estimated energy intake is < 60% of the required amount for a period of 1–2 weeks, it can result in calorie deficits, which leads to the exhaustion of body reserves [8, 9]. Increasing evidence confirms that malnutrition is associated with the underlying disease or inflammatory mechanisms; the latter generate anorexia, increased energy expenditure at rest and increased muscle catabolism [10]. Literature widely describes the negative effects of calorie–protein malnutrition on health outcomes [11, 12]. Fundamental in this treatment context is the analysis of the risk factors influencing this alteration during and after allo-HSCT [13–15]. Some studies show that 10–15% of patients are malnourished before transplantation. In addition, those with low body mass index (BMI) appear to be associated with a higher incidence of non-relapse mortality (NRM) as well as reduced overall survival (OS) [16]. On the other hand, many patients present themselves with a normal nutritional status at the initial diagnosis but with a real risk of significant weight loss during treatment. In the pre- and post-allo-HSCT phase, the nutritional status is potentially modifiable through support but often lacks standardized strategies; when and how to offer support remains a complex clinical decision for health professionals [17].

Although the effects of malnutrition on morbidity and mortality in adult allo-HSCT patients are well understood, no study investigated their relationship to quality of life (QoL) [18]. The purpose of this study was to evaluate the relationship between malnutrition and some aspects of QoL and to investigate the factors that contribute to reducing nutrient intake/absorption.

## Materials and methods

### Study design

A prospective observational study has been conducted collecting clinical and nutritional data from electronic medical records of enrolled patients over 18 years of age who underwent an allo-HSCT at our hospital in the period between May and December 2018, expressing their written informed consent to participate. The local ethics committee approved this study (protocol 46,787/17–1143/18, ID: 1767).

### Nutritional assessment

The Nutritional Risk Screening 2002 (NRS-2002) [19, 20] was used to perform nutritional screening to identify patients at risk as recommended by the European Society for Clinical Nutrition and Metabolism (ESPEN) guidelines [21, 22]. The adapted criteria of the Global Leadership Initiative on Malnutrition (GLIM) have been used, 14 days after infusion of the Hemopoietic Stem Cells (HSC) and on the day of discharge, for the diagnosis of malnutrition in clinical settings [23]:

DIAGNOSTIC ASSESSMENT (requires at least one phenotypic and one etiologic criterion):

Assessments criteria

- Phenotypic

ο Weight loss (%): > 5%;
ο Low BMI (kg/m^2^): ^<^20;
ο Phase angle (PA): Males < 5, Females < 4.6 [24];

- Etiological

ο Reduced food intake or assimilation: 50% of energy requirements (ER) > 1 week or any chronic gastrointestinal (GI) condition that adversely impacts food assimilation or absorption;
ο Disease burden/inflammation: acute disease/injury- or chronic disease-related, C-reactive protein (CRP);

Severity grading:

Severity of malnutrition determination

- Severity determined based on phenotypic criterion (requires one phenotypic criterion)

ο Moderate malnutrition: weight loss 5–10%; low body mass index < 20; moderate reduced muscle mass (validated assessment methods–see below);
ο Severe malnutrition: weight loss > 10%; low body mass index < 18.5; severe reduced muscle mass (validated assessment methods–see below).

Body weight was measured with a digital GIMA balance (to an accuracy of 0.1 kg); height measurement was carried out with an altimeter (approximately 0.5 cm). Phase angle is a well-established technique for diagnosing malnutrition and clinical prognostic tool [25, 26], and was measured through a tetra-polar detection system (BIA-AKERN). For the evaluation of food waste, a standardized detection methodology was adopted through the use of iconographies that identified the portioning of the dishes with the detection of consumption in percentage (0, 25, 50, 75, 100); such evaluation was conducted at each main and individual meal from day -2 to day + 14 of the allo-HSCT. Food intake was related to the estimated caloric intake for a standard hospital diet adapted to clinical needs by 2200 kcal (400 kcal at breakfast, 900 kcal at lunch and 700 kcal at dinner).

### Quality of life assessment

To assess key items of QoL such as energy levels, the ability to perform daily activities and the global patients’ perceiving of QoL, cancer linear analog scale (CLAS) is used. It is a linear analog self-assessment tool at 0 to 100 created to measure the effects and any benefits of anti-neoplastic treatments in patients. It is a simple, easy-to-use and easy-to-understand technique that allows you to monitor the impact of chemotherapy on the quality of life over time, providing reliable results with respect to perceived “well-being” [27, 28].

### Endpoint

The primary endpoint of the study was to assess the relationship between QoL and alteration of nutritional status in adult patients undergoing allo-HSCT. The CLAS was used at time of admission (T0), on the day of infusion of the HSC (T1), 7 and 14 days after infusion of the HSC (T2/T3) and on the day of discharge (T4).

### Sample size calculation

The sample size has been calculated considering an ANOVA mixed model with repeated measurements, with alpha = 0.01 and power 80% a delta = 0.6325; a variance between groups = 0.02 and a variance explained by effect “between–within groups” = 0.05 for 2 or more measurements repeated with a rho of correlation = 0.9 resulting in 36 participants required considering any dropouts.

### Statistical analysis

The sample has been described in its socio-demographic and clinical characteristics through descriptive statistical techniques. The qualitative variables were described using absolute frequencies and percentages, while the quantitative variables were synthesized through mean and standard deviation. Normal values were verified with the Skewness, Kurtosis and the Shapiro–Wilk *W* test. Comparisons were made with *t* test for non-addressed data or Kruskal–Wallis [29]. Data were stored and managed in spreadsheets (Data set built on spreadsheet type Microsoft Excel 2016 for Mac Vers. 2016/14.5.5). Statistical analysis was carried out through State/IC software [16.1 for Windows (64-bit Intel)]. The statistical significance was set at *p* 0.05.

## Results

We included 36 patients in our study. The proportion of missing data in the end of the study was minimal (< 5%). Patient characteristics, including information of transplantation performed, are shown in Table 1. Prophylactic parenteral nutrition was not given to our patients. Transplant-related mortality (TRM) was 14%, while the most frequent post-transplant complication was aGVHD (14%) followed by hemorrhagic cystitis (5%). The average time of admission was 50.7 days (SD 21.6), the average time of admission after allo-HSCT was 37.9 days (SD 21.7) and the average time to engraftment 18.16 days (SD 5.5) after allo-HSCT.

**Table 1:** General characteristics

	Freq	Percent	Cum
Age			
21–30	2	5.6	5.6
31–40	5	13.9	19.5
41–50	2	5.5	25.0
51–60	15	41.7	66.7
61–70	12	33.3	100
Gender			
Female	14	38.9	38.9
Male	22	61.1	100
Disease			
AML	12	33.3	33.3
MM/PCD	1	2.8	36.1
CLL	2	5.6	41.7
ALL	9	25.0	66.7
Ly	2	5.6	72.3
MDS/MPS	10	27.7	100
HSC source			
BM	17	47.2	47.2
PBSC	18	50.0	97.2
CB	1	2.8	100
Transplantation			
HLA Id. Sib	9	25.0	25.0
Unrelated Donor	10	27.8	52.8
Fam. Mismatch /Aplo	17	47.2	100
Conditioning			
TBF	30	83.3	83.3
CFM	2	5.6	88.9
BF	1	2.8	91.7
F	2	5.5	97.2
CF	1	2.8	100
TBI			
No	32	88.9	88.9
Yes, 12 Gy	3	8.3	97.2
Yes, 2 Gy	1	2.8	100
Therapeutic education			
No	18	50.0	50.0
Yes	18	50.0	100
Complications			
No	29	80.6	80.5
aGvHD	5	13.9	94.4
Hemorrhagic cystitis	2	5.6	100
TRM			
No	31	86.1	86.1
Yes	5	13.9	100
Alive			
No	8	22.2	22.2
Yes	28	77.8	100

*AML* acute myeloid leukemia; *MM/PCD* multiple myeloma/ plasma cells diseases; *CLL* chronic lymphocytic leukemia; *ALL* acute lymphocytic leukemia; *Ly* lymphoma; *MDS/MPD* myelodysplastic syndromes/myeloproliferative diseases; *BM* bone marrow; *PBSC* peripheral blood stem cell; *CB* cord blood; *HLA* human leukocyte antigen; *TBF* thiotepa busulfan fludarabine; *CFM* cyclophosphamide melphalan fludarabine; *BF* busulfan fludarabine; *F* fludarabine; *CF* cyclophosphamide fludarabine; *TBI* total body irradiation; *aGvHD* acute Graft-versus-Host Disease; *TMR* transplant-related mortality

### Nutritional status, support and causes of reduced nutrient intake/uptake

**Fig. 1:**
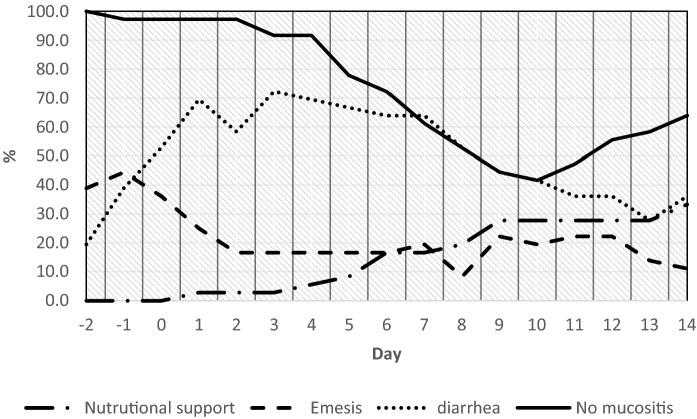
Relationship between complications and nutritional support during allo-HSCT

Body weight reduction of 5–10% or > 10% was recorded in 41.7% and 13.9% of patients respectively. In addition, the reduction of BMI < 20 involved 13.9% of the cohort (8.4% upon admission), while an altered phase angle was demonstrated in 66.7% (16.7% upon admission). Upon discharge, with the NRS, all patients were at risk (50% upon admission) and had moderate malnutrition (58. 3%), severe (13. 9%, Table 2). From day − 2 to day + 14 from allo-HSCT, the average albuminemia value was 27.5 g/L, and nutritional intake was on average 365 kcal. Figure 1 shows the use of nutritional support, the presence of emesis and diarrhea and the absence of mucositis.

**Table 2:** Nutritional status and risk

	Admission			Discharge		
	Freq	Percent	Cum	Freq	Percent	Cum
Body mass index						
20	33	91.7	91.7	31	86,1	86,1
< 20	2	5.6	97.3	4	11.1	97,2
< 18.5	1	2.8	100	1	2.8	100
Phase angle						
Altered	6	16.7	16.7	24	66.7	66.7
Normal	30	83.3	100	12	33.3	100
Nutritional risk screening						
3	18	50.0	50.0	36	100	100
< 3	18	50.0	100	0	0	100
Weight loss						
< 5%				16	44.4	44.4
5–10%				15	41.7	86,1
> 10%				5	13.9	100
Malnutrition						
No				10	27.8	27.8
Moderate				21	58.3	86.1
Severe				5	13.9	100.00

### Quality of life

Table 3 demonstrates results regarding QoL assessed with CLAS. The energy level was at admission 70.0 (95% CI 63.1–76.9) vs 48.9 (95% CI 40.7–57.1) at discharge (*p* = 0.01). The ability to carry out daily life activities was at admission 71.5 (95% CI 64.5–78.6) vs 46.2 (95% CI 37.2–55.2) at discharge (*p* = 0.01). The overall QoL was at admission 69.3 (95% CI 61.6–77.0) vs 46.0 (95% CI 37.0–55.10) at discharge (*p* = 0.01).

**Table 3:** Assessment of Quality of life during the allo-HSCT and at admission-discharge

Time	Obs	Mean	Std.err	Std.dev	95%CI		*P*–*v*
Energy							
T0	36	70.0		20.4			
T1	36	49.8		22.1			
T2	36	39.7		24.0			
T3	36	36.9		19.1			
T4	36	48.9		23.8			
T0	36	70.0	3.4	20.44	63.1	76.9	**0.01**
T4	36	48.9	4.0	23.83	40.7	57.1	
Ability to carry out daily life activities							
T0	36	71.5		20.8			
T1	36	49.2		23.2			
T2	36	39.4		25.2			
T3	36	36.6		19.5			
T4	36	46.2		26.1			
T0	36	71.5	3.5	20.9	64.5	78.6	**0.01**
T4	36	46.2	4.4	26.1	37.2	55.2	
Overall quality of life							
T0	36	69.3		22.9			
T1	36	40.3		23.3			
T2	36	36.9		25.6			
T3	36	34.1		23.3			
T4	36	46.0		26.4			
T0	36	69.3	3.8	22.9	61.6	77.0	**0.01**
T4	36	46.0	4.5	26.4	37.0	55.1	

Significant *p*-values are shown in bold

T0: admission; T1: allogeneic hematopoietic stem cell transplantation (allo-HSCT); T2: + 7 day after allo-HSCT; T3: + 14 day after allo-HSCT; T4: discharge

### Quality of Life and malnutrition

**Fig. 2:**
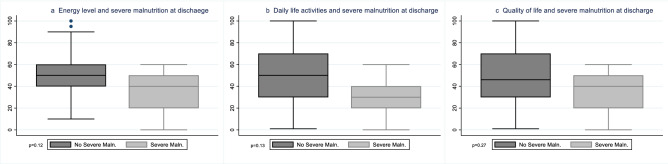
**a** Energy level and severe malnutrition at discharge. **b** Daily life activities and severe malnutrition at discharge. **c** Quality of life and severe malnutrition at discharge

**Fig. 3:**
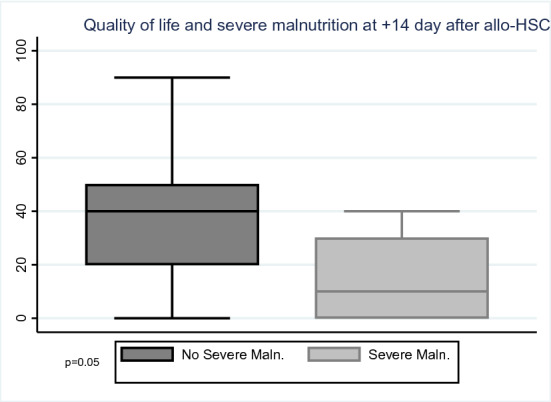
Quality of life and severe malnutrition at + 14 day after allo-HSCT

The *energy level* was 38.0 (95% CI 30.8–45.2) at 14 day after allo-HSCT in patients without severe malnutrition, 30.0 (95% CI 14.8–45.2) in patients with severe malnutrition (*p* = 0.38); 51.3 (95% CI 42.9–59.7) at discharge in patients without severe malnutrition, 34.0 (95% CI 4.1–63.9) in patients with severe malnutrition (*p* = 0.12), (Fig. 2a). The *ability to carry out daily life activities* was 38.3 (95% CI 31.2–45.5) at 14 day after allo-HSCT vs 26.0 (95% CI 7.2–44.8) (*p* = 0.18); 48.8 (95% CI 39.4–58.2) at discharge vs 30.0 (95% CI 2.2–57.8) (*p* = 0.13) (Fig. 2b). *Overall QoL* was 37.1 (95% CI 2.9–45.39) at 14 day after allo-HSCT vs 16.0 (95% CI − 6.6 to 38.6) (*p* = 0.05); 48.0 (95% CI 38.4–57.6) at discharge vs 34.0 (95% CI 4.1–63.9) (*p* = 0.27) (Figs. 2c; 3; Table 4).

**Table 4:** Relationship between Quality of life and severe malnutrition at discharge

	Time	Obs	Mean	Std.err	Std.dev	95%CI		*P*–*v*
Energy								
No severe malnutrition	*T3*	31	38.0	3.5	19.6	30.8	45.2	0.38
Severe malnutrition		5	30.0	5.5	12.2	14.8	45.2	
No severe malnutrition	*T4*	31	51.3	4.1	22.9	43.0	59.8	0.12
Severe malnutrition		5	34.0	10.8	24.1	4.1	63.9	
Ability to carry out daily life activities								
No severe malnutrition	*T3*	31	38.3	3.5	19.5	31.2	45.5	0.18
Severe malnutrition		5	26.0	6.8	15.2	7.2	44.9	
No severe malnutrition	*T4*	31	48.8	4.6	25.6	39.4	58.2	0.13
Severe malnutrition		5	30.0	10.0	22.4	2.2	57.8	
Overall quality of life								
No severe malnutrition	*T3*	31	37.1	4.0	22.5	2.9	45.3	**0.05**
Severe malnutrition		5	16.0	8.1	18.2	− 6.6	38.6	
No severe malnutrition	*T4*	31	48.0	4.7	26.2	38.4	57.6	0.27
Severe malnutrition		5	34.0	10.8	24.1	4.1	64.0	

Significant *p*-values are shown in bold

T3: + 14 day after allo-HSCT; T4: discharge

Comparing patients with mild/moderate malnutrition with severe malnutrition, the latter had lower QoL scale scores, even if not statistically significant (median “energy level “50 VS 30, “daily life activities” 50 vs 30, “overall QoL” 46 vs 40, respectively).

## Discussion

This study was conducted recruiting patients undergoing allogeneic HSCT, and, to our knowledge, we could not find published reports on the association of malnutrition and QoL in this setting. Our data suggest that at the time of discharge, transplanted patients exhibit a significant decrease of the levels of energy, of the ability to perform daily activities and in general of the QoL. Reduced QoL has been described in patients undergoing allografts, and may last several months, up to and over 1 year after HSCT [30, 31]; QoL is therefore recognized as an important marker to be considered when evaluating the safety and tolerability of allogeneic HSCT [32, 33].

Malnutrition in allogeneic HSCT has been associated with increased NRM, decreased survival and worse outcome [34, 35] Our data confirm that patients being discharged from the transplant unit, have a reduction of body weight and/or a BMI below lower normal value.

We have seen a trend, which does not reach largely statistical significance, for an association between lower body weight and worse QoL. Significant lower overall QoL in patients with severe malnutrition at 14 days after infusion of the HSC, needs to be confirmed in a larger group of patients. If this is the case, improvement of the nutritional status, may lead to improvement of the QoL, although one needs to take into consideration the multifactorial pathogenesis of malnutrition and of a poor QoL, to establish a correct cause/effect relation [36, 37] Enteral nutrition is the current recommended support, leaving parenteral nutrition for patients with reduced or impaired oral intake, due to mucositis, vomiting, or GvHD [38–41]; however, there are studies that describe parenteral fat emulsion is useful to maintain caloric intake without compromising glucose control after allogeneic HSCT [42]. We have studied the timing and factors influencing food intake in patients undergoing allogeneic HSCT: patients reduce food intake, including all nutrients, from the first days of the conditioning regimen, because of nausea and vomiting; in the following days, diarrhea is seen in 70% of patients, together with mucositis up until day + 9. At this time point, the majority of patients (72%) did not receive an adequate nutritional support. In addition, the supports were often delayed starting at a larger time after transplantation.

It is known that malnutrition predisposes patients to severe calorie and protein deficits which contribute, along with other factors, to muscle wasting and metabolic and functional deterioration. As demonstrated by the EFFORT study (Effect of Early Nutrition Support on Frailty, Functional Outcomes and Recovery of malnourished medical inpatients Trial), hospitalized patients at risk can take advantage of early (within 48 h of hospitalization) and individualized protein–calorie nutritional support, which can improve clinical outcomes, including survival [43]; it has been shown that this approach, compared to normal hospital nutrition, has brought about improvements in functional and QoL outcomes [44]. In conclusion, early malnutrition should be considered an expected event, and should call for early patient-tailored proactive intervention, with the aim to improve outcome.

## Limitations

The choice of the CLAS tool, during transplantation, was due to a simple design, which allowed easy compilation by the patients. These scales are able to provide scores of the considered items with the same “magnitude” of more complex tools for QoL assessment [45]. It should be noted that in the literature, among the different QoL evaluation tools, there has been little consensus on which tool should be used [46]

However, the use of an instrument that measures a characteristic that varies through a continuum of values, has limitations related to the difficulty for the patient to understand, and therefore in this case, to reduce bias, support was required during completion.

## Conclusion

Patients with major problems of malnutrition upon discharge, along with other complications associated with treatment (e.g., Infections, GVHD show a trend for worse QoL). Therefore, interventions to improve nutritional status might be beneficial to improve perceived QoL, as well as acting synergistically on clinical outcomes (OS, NRM, rooting, infections, etc.), which restores part of the lost energies and allows a better performance of daily activities. The maintenance of an adequate nutritional status that acts on the QoL may lead to significant benefits. However, to confirm this hypothesis, further prospective studies involving more patients are needed.

## Data Availability

The data that support the findings of this study are available from the corresponding author, [M.C.], upon reasonable request.
